# Reaction Enumeration Based on NBO‐Informed Molecular Graphs

**DOI:** 10.1002/jcc.70462

**Published:** 2026-07-06

**Authors:** Javier E. Alfonso‐Ramos, Thijs Stuyver

**Affiliations:** ^1^ Ecole Nationale Supérieure de Chimie de Paris Université PSL, CNRS, i‐CLeHS Paris France

## Abstract

Enumeration of reaction products and pathways is essential for understanding chemical reactivity and for the rational design of new chemical technologies. Traditional graph‐based enumeration methods represent molecules through atomic connectivity and simple valency rules, with bond breaking and formation encoded as edge modifications. However, this representation becomes ambiguous for many important classes of chemical systems, including transition‐metal complexes, hypervalent compounds, and molecules with extensive resonance or delocalization. Here, we introduce a molecular graph representation constructed at the level of valence orbitals rather than atomic connectivity alone. When combined with quantum‐mechanical natural bond orbital (NBO) analysis, the method automatically detects delocalized bonding motifs and adaptively increases reaction‐step complexity when chemically warranted. This integration enables systematic and computationally efficient identification of chemically meaningful reaction pathways that lie beyond the practical scope of conventional graph‐based approaches. We demonstrate the robustness of the method across challenging bonding environments, including organometallic catalytic cycles, pericyclic reactions in highly delocalized systems, main‐group hypervalent chemistry, and multicomponent reaction networks.

## Graphical abstract

NBO‐informed molecular graphs represent chemistry via valence orbitals rather than atomic connectivity, enabling consistent reaction analysis across environments. By identifying delocalized motifs and increasing complexity adaptively, the framework enables efficient exploration of inaccessible mechanisms and diverse catalytic and reaction networks for scalable reaction discovery and mechanistic analysis.

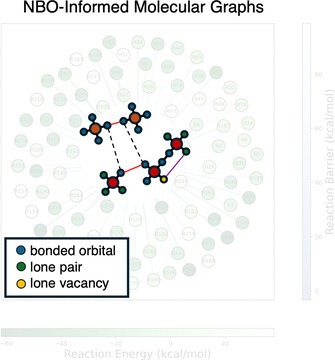

## 1 Introduction

Systematic, algorithmic exploration of chemical reactivity—in contrast to conventional, manual hypothesis generation carried out on a trial‐and‐error basis—has long been a central aspiration in computational chemistry. Early conceptual foundations were laid by Ugi and Dugundji, who introduced the use of bond and electron matrices to encode molecular systems and proposed reaction matrices as a formal means of describing their interconversions [1, 2]. These early ideas already contained the seeds of an automated, rules‐based exploration of chemical space.

With the steady expansion of computational power over the last few decades, increasingly sophisticated algorithms have been developed to practically realize this vision [3, 4]. Contemporary methods such as the artificial force‐induced reaction (AFIR) approach [5, 6], molecular “nanoreactors” [7, 8, 9], and the recently developed Chemoton framework [10, 11, 12], seek to enumerate reaction pathways by driving systems over potential‐energy barriers/bottlenecks, or by recursively identifying new intermediates and transition states starting from high‐energy reactive complexes. A key strength of these methods is their intentional minimization of heuristic assumptions: they attempt to let the underlying potential energy surface speak for itself, enabling relatively unbiased discovery of both expected and unexpected mechanistic routes. However, this reduction in bias comes at a substantial computational cost. Even with modern hardware, these approaches typically demand extensive sampling and repeated electronic‐structure calculations, which restricts their application to relatively small, or carefully curated, systems.

Graph‐based strategies for reactivity exploration offer a complementary—and often more tractable—alternative. By adopting a molecular graph representation of reactants, they provide a natural way to encode bonding patterns, valency rules, and functional‐group transformations. This abstraction enables efficient traversal of large chemical spaces and the rapid generation of plausible elementary transformations, which can subsequently be confirmed with an appropriate electronic structure approach. A wide variety of graph‐based mechanistic exploration strategies have emerged, spanning stochastic [13, 14], rule‐based [15, 16], and data‐driven enumeration approaches [17].

Recent graph‐based enumeration frameworks have employed different definitions of elementary reaction complexity depending on the underlying graph representation. In bond‐based formalisms, elementary steps are typically characterized by the number of bonds broken and formed in a single transformation, whereas valence‐ or electron‐based approaches instead describe reactions through redistribution of valence electrons or orbital occupations [18, 19, 20]. These alternative representations can lead to substantially different scaling behavior and notions of mechanistic complexity for the same chemical transformation. Bond‐based representations provide an explicit specification of reactant and product connectivity, which simplifies construction of chemically valid product graphs, whereas valence‐ or electron‐based approaches encode the underlying electron redistribution more directly but may require additional procedures to reconstruct unambiguous bonding patterns in the products. For this reason, bond‐based representations have become particularly widespread in practical reaction‐network enumeration frameworks, where explicit generation of chemically consistent product structures is essential for efficient exploration.

Recent work by Savoie and co‐workers [21, 22, 23], in particular, has demonstrated how graph models and reaction‐network formalisms can be used in practice to elucidate mechanistic landscapes with manageable computational effort. Their central idea is to systematically enumerate possible elementary steps while imposing a strict upper bound on their complexity. Most commonly, this bound is expressed in terms of the maximum number of bonds broken and formed in a single step, with a limit of two bonds broken and two bonds formed (b2f2) serving as the default choice in many of their studies.

Although such constraints are essential to curb the combinatorial explosion of possible pathways, it is well established that b2f2 moves are overly restrictive for many areas of chemistry and can exclude crucial mechanistic steps. A canonical example is the Diels–Alder reaction, which is formally a b3f3 transformation and would therefore not appear in a purely b2f2‐based analysis. Recognizing this limitation, several recent efforts have sought to relax the b2f2 constraint in targeted chemical domains. Notably, Casetti and co‐workers have explored up to b4f4 transformations as part of a pipeline for prospectively detecting plausible cyclization reactions in conjugated organic systems [24]. As expected, expanding beyond b2f2 dramatically increases the number of potential products to be considered—often by more than two orders of magnitude, even for fairly small systems—highlighting both the promise and the computational challenges of more general graph‐based reactivity exploration.

A natural strategy to better balance tractability with completeness would be adaptive modulation of allowed reaction‐step complexity, retaining b2f2 moves in general but permitting more complex transformations whenever chemically justified. A key requirement for such an adaptive scheme is the ability to identify electronic delocalization, or other bonding motifs that enable multi‐bond rearrangements. For conventional organic molecules, this is relatively straightforward: alternating single and double bonds or other conjugated motifs can be readily recognized from SMILES strings or standard graph representations. However, for many classes of systems—including dipolar structures, hypervalent compounds (e.g., involving S or P), and especially organometallic complexes—this cannot be predicted a priori. Furthermore, a single, unambiguous Lewis structure often does not even exist for these systems. Constructing a molecular graph that truly reflects the bonding situation from a SMILES string is then impossible, which not only complicates the identification of delocalization, but also renders the entire enumeration process of plausible bonding rearrangements as a whole error‐prone.

In this work, we introduce a new open‐source graph‐based reaction‐pathway enumerator, designed to address precisely this challenge. In addition to an option for constructing traditional, electronic‐structure‐agnostic molecular graphs directly from SMILES‐derived connectivity, our software provides an alternative workflow in which graph generation is coupled to natural bond orbital (NBO) analysis [25]. As previously highlighted by Balcells et al. within the context of metal complexes [26, 27], because NBO outputs explicitly encode electron delocalization, hyperconjugation, and multi‐center bonding interactions, they offer a principled, data‐driven foundation for constructing molecular graphs in cases where conventional approaches become ambiguous or outright fail. Leveraging these NBO‐derived graphs, our framework can automatically detect delocalized motifs and adaptively expand the allowable reaction‐step complexity whenever chemically justified. This enables a more targeted and chemically informed expansion of the reaction space, particularly for systems with extensive or atypical delocalization. As a result, we can substantially extend the scope of mechanistic exploration‐for example, by uncovering auto‐ and solvent‐catalytic pathways that would never appear in a traditional b2f2, or even b3f3, analysis.

To demonstrate the scope and capabilities of our enumerator, we first benchmark its performance on the comprehensive organic reaction pathway dataset of Zimmerman and co‐workers [18, 28], assessing its ability to recover the registered products. We then illustrate how the integration of NBO‐derived delocalization signatures enables the efficient identification of organic reaction pathways that lie far beyond the reach of conventional b2f2‐based enumeration. Subsequently, we highlight the method's ability to recover organometallic catalytic cycles and to generate alternative mechanistic hypotheses in these more complex bonding environments. Finally, we couple our enumerator with our previously developed in‐house TS‐tools software [29] for automated transition‐state localization, enabling a fully automated reaction‐network exploration workflow.

As a final note, we would like to highlight that while the current implementation incurs an overhead associated with performing NBO calculations, we view this primarily as an engineering constraint rather than a conceptual limitation. Indeed, recent advances in machine‐learned NBO graph prediction [30] suggest a clear path toward eliminating this bottleneck. By integrating such predictive models, future versions of our approach could retain the chemical meaningfulness of NBO‐derived graphs while achieving near‐instantaneous graph construction.

## 2 Methods

This section outlines the overall structure of the reaction‐pathway enumerator. We first describe the construction of molecular graphs in terms of valence orbital (VO) arrangements (Section 2.1). We then summarize the procedure used to enumerate elementary reaction steps based on these graphs (Section 2.2). Finally, we present the computational details relevant to the reaction characterizations considered in the case studies (Section 2.3).

### 2.1 VO Graph

In our framework, molecular graphs are formulated at the level of VOs rather than atomic connectivity alone. Two fundamental entities are introduced: VO and localized orbital system (LOS). Each VO may be empty, singly occupied, or doubly occupied, and can either remain localized on a single atom or participate in bonding. LOS are constructed by grouping one or more VOs into chemically meaningful units such as lone pairs, vacancies, or bonding interactions.

Graph construction begins by representing atoms as nodes and generating their associated VOs. For p‐block elements, four VOs are included (one s‐type and three p‐type), whereas for d‐block elements, nine VOs are considered (one s, three p, and five d orbitals). In the electronic‐structure‐agnostic mode of the software (*vide supra*), the number of valence electrons is obtained from RDKit and distributed among the VOs according to the Pauli exclusion principle and Hund's rule.

**FIGURE 1:**
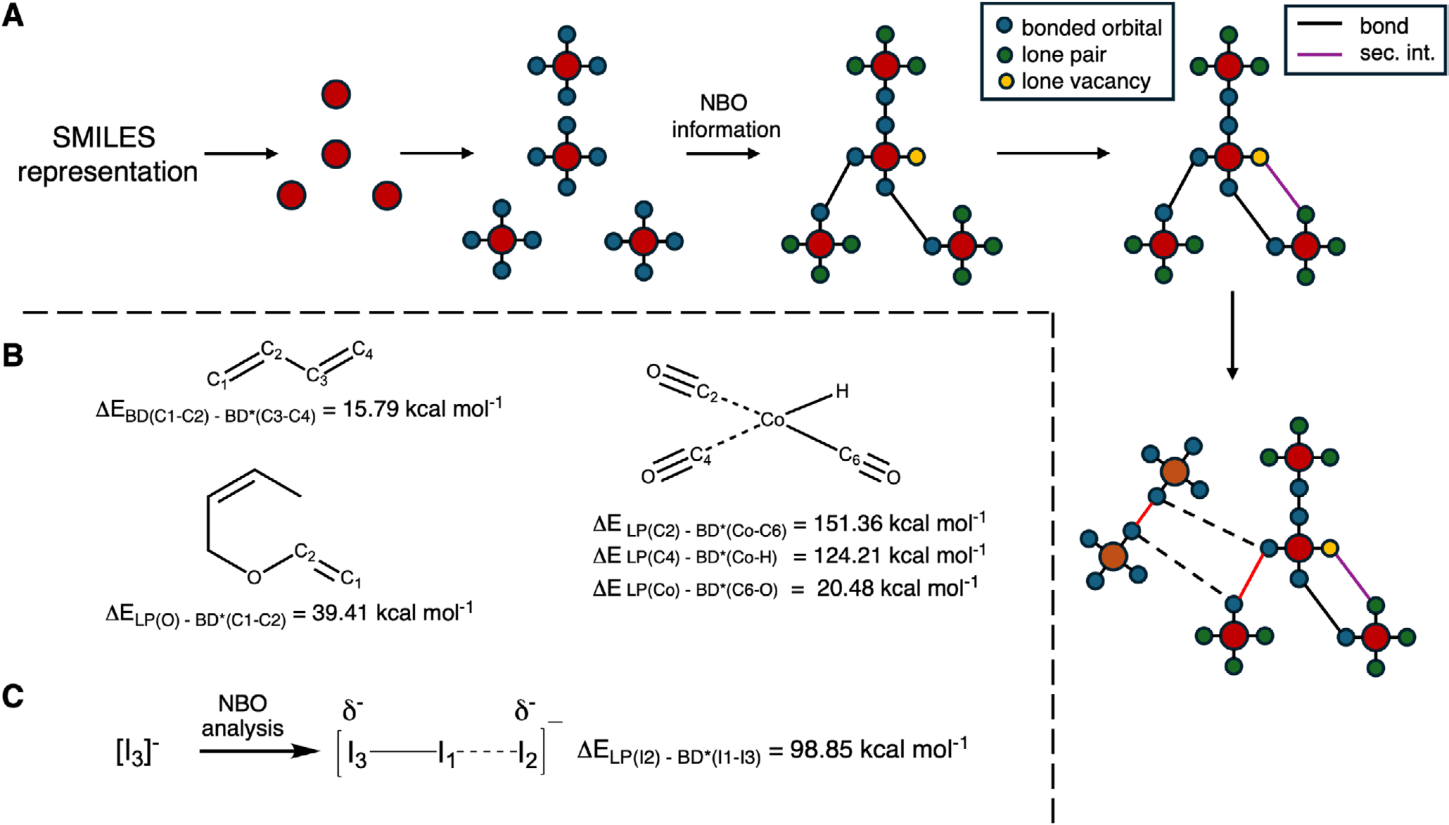
(A) Starting from SMILES strings for each initial reactant, NBO‐based graphs are systematically constructed in a set of steps: first atom nodes are defined, then valence orbitals (VO) associated with these atom nodes are initialized, after which all generated VOs are characterized as lone pairs/vacancies or bonded orbitals with help of computed NBO information. Once the graphs are fully constructed, reactions can be enumerated by considering VO permutations between reaction partners. (B) Examples of molecules exhibiting electron delocalization and the magnitude of secondary interactions. The cobalt complex is depicted according to the NBO analysis, showing two covalent bonds (solid lines) and two strongly stabilizing donor–acceptor interactions (dashed lines). (C) NBO representation of the triiodide anion. BD denotes a bonding orbital, LP a lone pair orbital, BD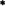 an antibonding orbital, and *sec. int.* a secondary interaction.

Following VO initialization, the LOSs are constructed. In the electronic‐structure‐agnostic mode, VOs that are either empty or doubly occupied are first identified and used to form LOSs, which are classified as empty valences or lone pairs, respectively. Next, singly‐occupied VOs are examined. For each such VO, a search is performed for a complementary singly occupied VO on a bonded neighboring atom, and the two orbitals are paired to form a bonding LOS (cf. Figure 1A). These bonding LOSs define the edges of the molecular graph and are consistent with the connectivity encoded in the SMILES representation. Singly occupied VOs for which no bonding partner is found are kept as radical sites.

As such, once the graph construction is completed, a set of LOSs is defined, each of which consists either of a single VO (corresponding to a lone pair, a vacancy, or a radical site) or of two VOs forming a σ‐ or π‐bond. The resulting graph corresponds to a conventional Lewis‐like representation and is suitable for systems where bonding patterns are unambiguous.

As an alternative to SMILES‐derived connectivity, the same valence‐orbital graph can be constructed using information obtained from NBO analysis [25]. NBO analysis transforms a given computed delocalized wavefunction into a localized description, consisting of an orthonormal set of localized orbitals that closely resemble a Lewis‐type bonding picture. In this procedure, atomic orbitals are first transformed into natural atomic orbitals, which are subsequently combined to form natural bond orbitals‐namely, bonding and antibonding orbitals, as well as lone pairs and vacancies. Importantly, this representation is largely independent of the underlying atomic basis set. In addition to identifying localized orbitals, NBO analysis provides quantitative information on donor‐acceptor interactions through second‐order perturbation analysis (SOPA), capturing, and quantifying, effects such as π–π conjugation, hyperconjugation, and multicenter bonding.

The construction of the molecular graph using NBO data follows a procedure similar to that employed with RDKit, with two notable differences. First, the valence electron count for each atom is obtained directly from the NBO results. Second, the construction of the LOSs is determined from the NBO analysis, rather than from the predefined SMILES connectivity.

Crucially, stabilizing donor–acceptor interactions identified in the SOPA analysis can be used to detect and represent electronic delocalization. Orbitals participating in such interactions are grouped into delocalized orbital systems by introducing secondary edges in the molecular graph (Figure 1A). This explicit representation of delocalization provides the basis for identifying bonding motifs that can support concerted, multi‐bond rearrangements in subsequent reaction‐pathway enumeration in a rigorous, and principled, manner.

Of course, not all donor‐acceptor interactions identified in the SOPA output imply a chemically meaningful degree of concerted electron motion. For example, in methylamine, the nitrogen lone pair interacts with an antiperiplanar C–H $\sigma^{*}$ orbital with a stabilization energy of 9.41 kcal mol^–1^ at the PBE0/def2‐SVP level of theory. While this interaction is clearly detectable at the orbital level, it would be unreasonable to interpret it as evidence that the two corresponding LOSs are sufficiently coupled to systematically participate jointly in chemical reactivity.

By contrast, in extended conjugated systems—where concerted electron rearrangements are well established—SOPA typically assigns substantially larger stabilization energies. Canonical π–π interactions commonly exhibit stabilizations on the order of 15 kcal mol^–1^, while hyperconjugative n‐π interactions can reach values of approximately 40 kcal mol^–1^ (cf. Figure 1B). These larger interaction energies are indicative of a qualitatively different regime, in which multiple orbitals are strongly coupled and can therefore be regarded as a unified, delocalized electronic subsystem.

To distinguish such chemically meaningful interactions from weaker, incidental ones, our implementation employs an energy‐based cutoff on the SOPA stabilization values. Only donor–acceptor interactions exceeding this threshold are used to define delocalized orbital systems and to introduce secondary edges in the molecular graph. By default, this cutoff is set to 12.00 kcal mol^–1^ throughout this work (unless stated otherwise), reflecting a conservative separation between weak hyperconjugative effects and interactions that plausibly support concerted bond rearrangements. This criterion is referred to throughout the remainder of this work as *threshold‐sec‐int*. Importantly, this parameter is fully adjustable by the user of our software package, enabling straightforward adaptation to different chemical domains or levels of electronic structure theory.

For transition‐metal complexes (TMCs), the electronic structure of the metal center and the way it is resolved within NBO analysis necessitates the introduction of a second energetic threshold in order to recover a chemically complete bonding network in the molecular graph. In contrast to typical main‐group systems, metal‐ligand bonding in TMCs is frequently distributed over multi‐center interactions that are not always represented as explicit two‐center bonds in the NBO output.

A representative example is provided by [Co(H)(CO)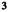] (Figure 1B), a key intermediate in cobalt‐catalyzed hydroformylation. In this case, the NBO analysis reports only two explicit cobalt‐ligand bonds: one to the hydride and one to a single carbonyl ligand. Taken at face value, this description would imply a severely undercoordinated metal center. However, inspection of the SOPA data reveals two additional, extremely strong donor–acceptor interactions, each exceeding 120 kcal mol^–1^ (dashed lines in Figure 1B). These interactions originate from donation of the carbonyl carbon lone pair into antibonding Co–H and Co–C orbitals, respectively. Taken together, the two‐center bonds and the strongly interacting (doubly occupied) mono‐center orbitals are most appropriately described as three‐center, four‐electron hyperbonds.

To ensure that such chemically essential interactions are incorporated into the molecular graph, we therefore introduce a second, higher energetic threshold that is applied specifically to capture these strongly stabilizing, metal‐centered donor–acceptor interactions. Unlike the lower cutoff used to identify delocalized valence systems in regular main‐group chemistry, the appropriate value of this higher threshold is highly system‐dependent: Our explorations so far indicate that the magnitude of these stabilizations varies with the electronic structure of the metal center, the nature of the ligands, and the overall coordination environment. Nonetheless, introducing this second criterion is crucial for constructing molecular graphs that faithfully represent the bonding topology of TMCs and for enabling meaningful reaction enumeration in organometallic systems. This second criterion is referred to throughout the remainder of this work as *threshold‐strong‐sec‐int*.

Importantly, this second energetic threshold is not unique to transition‐metal chemistry, but also plays a role in other exotic bonding situations that lie outside classical valence heuristics, most notably main‐group hypervalency. A prototypical example is the triiodide anion, I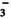. Although the bonding in such molecules has long been debated [31, 32, 33], the NBO framework provides a consistent description in terms of three‐center, four‐electron (3c–4e) hyperbonds, which account for the apparent violation of simple valency rules. By explicitly representing these interactions, our approach enables unambiguous electron bookkeeping and, as a consequence, the automated generation of chemically meaningful reaction pathways for systems exhibiting hypervalent or otherwise nonclassical bonding.

Figure 1C illustrates this representation: the negative charge is delocalized over the two terminal iodine atoms, only a single conventional bond is identified (solid line), and the third iodine atom is connected to the system via a 3c–4e hyperbond (dashed line).

These types of multicenter interactions, which are present in TMCs and hypervalent compounds, are initially treated in a manner analogous to secondary interactions by grouping the corresponding orbitals into delocalized orbital systems. However, during the product‐enumeration procedure, they are subsequently regarded as proper bonds.

A diagram summarizing the key steps involved in constructing the VO graphs is provided in Section S1.

### 2.2 Reaction Pathway Enumeration Procedure

Reaction‐pathway enumeration in our framework is formulated as a systematic redistribution of VOs between reacting species. The procedure begins by defining a set of active LOSs. By default, all LOSs that are not “chemically redundant” are considered active. By redundant LOSs, we mean symmetry‐equivalent bonding situations that would, in most cases, generate duplicate reaction hypotheses‐for example, the second and third C–H bonds in a methyl group, for which only a single representative C–H bonding LOS is retained. Analogously, only one LOS is retained for double and triple bonds. These defaults can be modified by the user, allowing for a more restrictive selection of active orbital systems when the exploration is intended to focus on a narrow subset of mechanistic possibilities.

Once the active LOSs have been identified, reaction pathways are generated in two stages: *intra‐fragment*path construction and *inter‐fragment* path assembly.

Intra‐fragment paths correspond to bonding rearrangements confined to a single molecular fragment. These paths are constructed by initiating from the VOs of a selected LOS, and optionally, recursively extending the path with VOs belonging to neighboring LOSs, until a predefined maximum path length is reached. The intra‐fragment path length is defined by the number of VOs it contains, and the maximum path length used to terminate the path extension is set to two VOs by default (this parameter can be adjusted by the user). During this process, several constraints are enforced to ensure chemically meaningful paths: orbitals already included in a given path are excluded from further extension, and non‐crossing conditions are imposed to prevent unphysical rearrangements within a fragment.

When NBO data are available, secondary donor‐acceptor interactions identified in the SOPA analysis are incorporated at this stage. Specifically, donor and acceptor LOSs participating in significant stabilizing interactions are mapped onto their corresponding VOs and grouped directly into intra‐fragment paths representing delocalized electronic systems. For example, in 1,3‐butadiene the two π bonds can be combined into a single delocalized path containing four VOs, while in 1,3,5‐hexatriene the extended π system yields a six‐VO path, even when the maximal intra‐fragment path length is set to two.

In a second step, the intra‐fragment paths generated for each reactant are exhaustively combined to form inter‐fragment paths, which describe VO rearrangements spanning all reacting species. Before bond rearrangements are applied, the algorithm determines whether explicit electron redistribution is required. For covalent and radical rearrangements, no redistribution is necessary, as each VO contributes one electron. In contrast, processes such as nucleophilic attack involve the transfer of an electron from a donor orbital (e.g., a lone pair) into an acceptor orbital associated with a leaving group. In such cases, VO occupancies are updated accordingly, and the formal charges of the affected atoms are adjusted to maintain electron‐count consistency. Finally, the terminal VO of the path is examined to determine whether it should be connected to the initial VO, thereby closing the path when required. For intramolecular reactions, an intramolecular path is constructed in an analogous manner by combining two intra‐fragment paths.

Based on the VOs present in each inter‐fragment path, the molecular bonding network is modified using the editable molecule object implemented in RDKit. For each pair of consecutive VOs along the path, the presence of an existing bond is evaluated: if a bond exists in the molecule object, it is broken; if no bond is present, a new bond is formed. Finally, the terminal VO of the path is examined to determine whether it should be connected to the initial VO, thereby closing the path when required. The resulting modified molecular object is converted back into SMILES representations, after which RDKit is used to exhaustively enumerate all corresponding stereoisomers, whenever appropriate.

**FIGURE 2:**
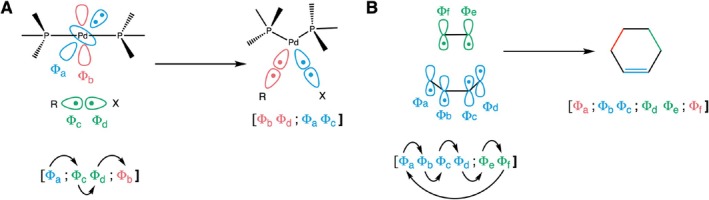
Two representative applications of the enumeration protocol. (A) Concerted oxidative addition between a palladium complex and an alkyl halide. The inter‐fragment path (between brackets) comprises a Pd lone pair, the two VOs of the C–X bond, and a vacant Pd orbital. After electron redistribution, bonds are formed or broken by evaluating interactions between consecutive VOs along the path. (B) Diels–Alder cycloaddition, where the inter‐fragment path is constructed from secondary π‐type interactions identified via NBO analysis. Existing π bonds are broken and new C–C bonds are formed following the same VO‐based protocol, with closure of the path completing the cycloaddition.

Figure 2 illustrates two representative applications of the enumeration protocol. The first example corresponds to a concerted oxidative addition between a palladium complex and an alkyl halide. Because this transformation involves an organometallic species, the inter‐fragment path comprises a lone pair on the Pd center, the two VOs associated with the C–X bond of the alkyl halide, and a vacant orbital on palladium. The enumeration begins with electron redistribution: one electron is transferred from $\Phi_{a}$ to $\Phi_{b}$ to enable subsequent covalent bond formation. The algorithm then evaluates interactions between consecutive VOs along the path. Since $\Phi_{a}$ and $\Phi_{c}$ belong to different fragments and are not initially bonded, a new bond is formed. The interaction between $\Phi_{c}$ and $\Phi_{d}$ corresponds to the original C–X bond and is therefore broken. Finally, a new bond is formed between $\Phi_{d}$ and $\Phi_{b}$. The terminal‐initial VO connection is skipped in this case, as both orbitals reside on the same atom and are already involved in newly‐formed bonding interactions. This procedure yields the oxidative addition product.

The second example (Figure 2B) corresponds to a Diels–Alder cycloaddition. Here, the inter‐fragment path is constructed using secondary interactions identified from the NBO analysis. The VOs $\Phi_{a}$–$\Phi_{b}$ and $\Phi_{c}$–$\Phi_{d}$ represent the two π bonds of the diene, while two additional VOs describe the π bond of ethylene. The same bond‐evaluation procedure is applied: the original π bonds are broken, and new bonds are formed between $\Phi_{b}$–$\Phi_{c}$ and $\Phi_{d}$–$\Phi_{e}$. In this case, the terminal and initial VOs must be connected to close the reaction path and complete the cycloaddition. Notably, the same inter‐fragment path can also be generated without relying on NBO‐derived secondary interactions.

### 2.3 Computational Details

In our enumerator software, the VO graph can be constructed directly from the SMILES representation of the molecular structures [34, 35]. When NBO‐based graph construction is employed, the lowest‐energy conformer of each molecule is first identified using autodE in combination with GFN2‐xTB [36, 37]. This conformer is subsequently used as the input geometry for the NBO analysis. All NBO calculations are performed using Gaussian16 interfaced with NBO 7.0 [38, 39]. Unless stated otherwise, all density functional theory calculations reported in this work are carried out at the PBE0/def2‐SVP level of theory [40, 41]. A sensitivity analysis, demonstrating the robustness of the NBO‐determined interactions to both changes in terms of functional and basis set can be found in Section S2 of the ESI.

To reduce the number of chemically implausible reaction pathways prior to more expensive electronic‐structure analysis, a crude, yet rapid, thermodynamic screening step is applied. Starting from the SMILES representations of reactants and products, initial conformers are generated using RDKit and optimized with xTB [37, 42], after which reaction energies are evaluated. Pathways associated with reaction energies exceeding 50 kcal mol^–1^ are discarded. This filter is not intended to provide quantitative thermodynamic accuracy, but rather to eliminate obviously unfeasible transformations and focus subsequent analysis on energetically reasonable candidates.

For selected reaction pathways, kinetic feasibility is assessed by computing full reaction profiles using a development version of TS‐tools using default parameters [29]. A detailed description of the transition‐state search protocol is provided in Section S3 of the ESI.

The results presented in this study were obtained using a VO graph representation enriched with NBO information. The default parameters include a cutoff of 12.00 kcal mol^–1^ for including a stabilization interaction as a secondary edge (*threshold‐sec‐int*) and a cutoff of 85.0 kcal mol^–1^ for identifying strongly stabilizing secondary interactions (*threshold‐strong‐sec‐int*). The maximum length of an intra‐fragment path is set to two VOs. In contrast, inter‐fragment paths are generated by combining one inter‐fragment path from each fragment, without restrictions on the number of VOs involved. Intramolecular paths are constructed via the combination of two inter‐fragment paths. These default settings are used throughout the remainder of this paper unless a different setup is explicitly specified.

Section S5 of the ESI provides guidelines for selecting appropriate threshold values when exploring different chemical systems, while Section S4 presents a sensitivity analysis demonstrating the robustness of the default thresholds for representative examples of both main‐group and organometallic systems.

As stated above, our algorithms account for the enumeration of all possible stereoisomers from a given SMILES representation. This option was disabled in only two cases. First, during the evaluation of the benchmarking set, the goal was to assess the chemical transformations that our algorithm is able to recover and to enable direct comparison with previous methods that do not include stereochemical enumeration of products. Second, for organometallic reactions, stereochemical enumeration was disabled because RDKit, at its current stage of development, cannot reliably handle stereochemistry in TMCs.

## 3 Results and Discussion

### 3.1 Benchmarking Set

To benchmark the performance of our reaction‐pathway enumeration approach, we employed a curated version of the Zimmerman dataset [18, 28]. Although this dataset was originally introduced to evaluate the performance of the growing string method (GSM) in transition‐state searches, it has subsequently been adopted as a reference benchmark for single‐step reaction enumeration [23]. The subset considered here comprises 30 unique reactants, giving rise to a total of 85 products (see Section S7 of the ESI). Both neutral and charged closed‐shell species are included.

Using the default maximum intra‐fragment path length of two VOs, our approach successfully recovers 62 of the 85 products, both with and without the inclusion of NBO‐derived information. This corresponds to a success rate of approximately 73%, which is consistent with the performance reported for b2f2‐based enumeration in the YARP framework developed by Savoie and co‐workers. Increasing the maximum number of VOs per intra‐fragment pathway to four leads to the recovery of 66 products, corresponding to an improved success rate of 79%.

**FIGURE 3:**
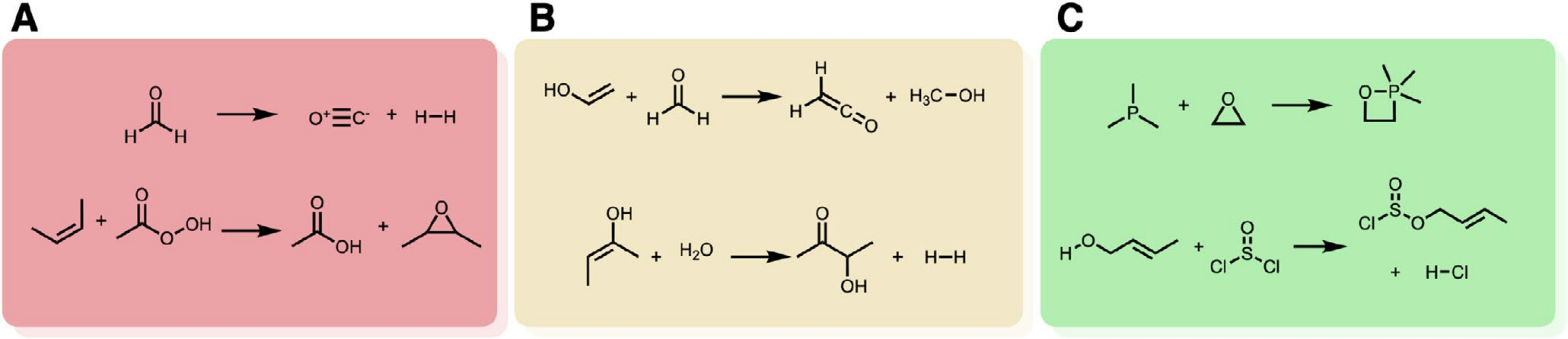
(A) Representative examples of reaction types not recovered by the current enumeration protocol. (B) Chemical reactions that can be recovered when up to four valence orbitals are allowed within each inter‐fragment path. (C) Transformations that we successfully recover but remains outside of the scope of b2f2 formalism.

The primary source of failure can be traced to chemical transformations that formally require the simultaneous formation or cleavage of two bonds at the same main‐group atom. As discussed above, such transformations are deliberately excluded in our enumeration scheme, as they are typically associated with prohibitively high kinetic barriers and are therefore unlikely to correspond to chemically viable elementary steps. For instance, the first reaction in Figure 3A exhibits an activation energy of 81.71 kcal mol^–1^, and the Cl_2_ elimination of SO_2_Cl_2_ in the Zimmerman dataset presents a barrier of 80.00 kcal mol^–1^. One notable example of a transformation that is arguably excluded too conservatively is epoxidation (cf. the second example in Figure 3A presents an activation energy of 21.84 kcal mol^–1^), which involves concerted formation of two bonds at a single atom yet is known to proceed with accessible barriers in many cases.

Importantly, however, the realism or feasibility of individual reaction pathways was not a selection criterion in the original construction of the Zimmerman dataset. As a result, some reactions included in the benchmark fall outside the intended scope of our method.

Figure 3B illustrates representative reactions that are recovered when the maximum intra‐fragment path length is increased to four VOs, but not when it is restricted to two, underscoring the benefits of allowing moderately more complex orbital rearrangements within a controlled enumeration framework. Finally, Figure 3C shows two examples of reactions that are recovered by our algorithm but lie outside the b2f2 formalism. We highlight the first example in particular, as it demonstrates that by properly treating hypervalency during enumeration, one can recover reaction pathways that would not naturally emerge through a naive valency treatment.

### 3.2 Delocalization‐Enabled Enumeration of Pericyclic Reactions

We now turn to a class of transformations for which the explicit inclusion of NBO‐derived delocalization information provides a clear and tangible advantage. Pericyclic reactions are ubiquitous in organic chemistry and constitute a versatile and widely exploited family of transformations for the synthesis of carbo‐ and heterocycles. When classified in terms of bond‐breaking and bond‐forming events, these reactions frequently fall into the b3f3 category or beyond. As a result, exhaustive product enumeration rapidly becomes computationally prohibitive as molecular size and substitution patterns increase.

By explicitly embedding π‐conjugated systems into the VO graph, our approach naturally captures the concerted nature of pericyclic rearrangements. In particular, the π system of the diene is represented as a single delocalized orbital path, allowing the main [4+2] cycloaddition channel—and even higher—order pericyclic processes‐to be recovered at the shortest possible intra‐fragment path length. Crucially, this is achieved without incurring the intrinsic combinatorial overhead associated with conventional b3f3 or b4f4 enumeration schemes.

**FIGURE 4:**
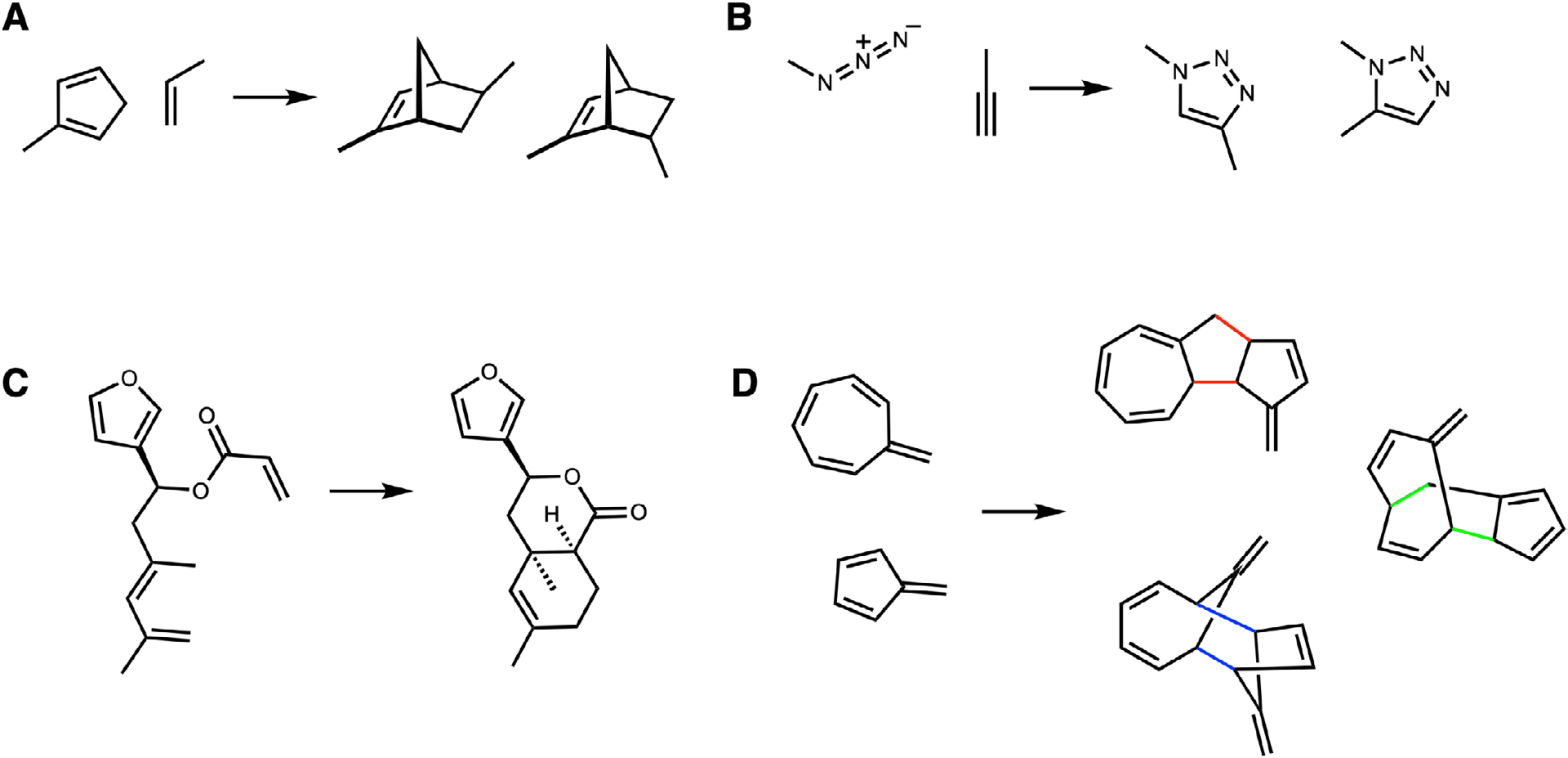
(A) Typical [4+2] Diels–Alder reaction between a substituted cyclopentadiene and propene. (B) Click (3+2) cycloaddition reaction between methyl azide and propyne. (C) Key step during the synthesis of Salvinorin A. (D) 3 possible cycloaddition products ([6+4], [4+6], [8+2]) between tropane and fulvene.

As a first illustrative example, we consider the Diels–Alder reaction between 2‐methylcyclopent‐1,3‐diene and propene. Using our algorithm, a total of 94 products are enumerated at a maximum intra‐fragment path length of two. After exhaustive stereoisomer generation, this corresponds to 300 distinct reaction pathways, including both regioisomeric [4+2] cycloaddition products. Figure 4A highlights the relevant Diels–Alder adducts; for clarity, only one representative stereoisomer of each product is shown.

A second example is provided by the (3+2) cycloaddition between methyl azide and propyne, yielding a triazole product [43, 44]. Although this transformation formally belongs to the b2f2 class, it lies outside the practical scope of many previous enumeration approaches due to its charged character and the presence of multiple competing resonance structures. Applying our protocol, an unambiguous molecular graph is constructed, enabling the enumeration of 76 potential products (Figure 4B). When all possible stereoisomers are taken into account, this number increases to 104.

Moving beyond model systems, we next examine two chemically relevant cases. A key step in the synthesis of the natural product salvinorin A involves an intramolecular Diels–Alder reaction (Figure 4C). A recent study reported that more than 23,000 products (accounting for all the possible stereoisomers) must be considered to exhaustively enumerate this transformation [24]. By contrast, exploiting NBO‐derived delocalization information, our approach recovers the correct cycloadduct using default settings while requiring the enumeration of only 5699 products—representing a reduction by approximately a factor of four. This figure reflects exhaustive enumeration over stereoisomers and distinct reaction pathways; when this distinction is not considered, only 795 potential products need to be enumerated.

As a final example, we consider the cycloaddition between a tropone derivative and fulvene (Figure 4D), which can, in principle, proceed via three distinct modes: [6+4], [4+6], and [8+2] [45]. Exhaustive sampling of these possibilities would require at least a b5f5 enumeration, leading to tens of thousands of candidate products. Instead, by leveraging secondary orbital interactions identified through NBO analysis, our method recovers all chemically relevant cycloadducts while enumerating only 255 products in total, corresponding to 3464 stereoisomers and distinct reaction pathways.

### 3.3 Efficient Enumeration of Reactivity in Trimolecular Systems

A further application of the present methodology—one that lies beyond the reach of naive b2f2 enumeration schemes—is the treatment of tri‐ and multicomponent systems, such as solvent‐assisted or autocatalyzed processes. To illustrate the capability of our approach, we consider the reaction between formaldehyde and vinyl alcohol in the presence of a single water molecule acting as a catalyst [5]. Using our enumeration protocol, a total of 77 products are generated, of which 68 pass the coarse thermodynamic screening threshold of 50 kcal mol^–1^.

**FIGURE 5:**
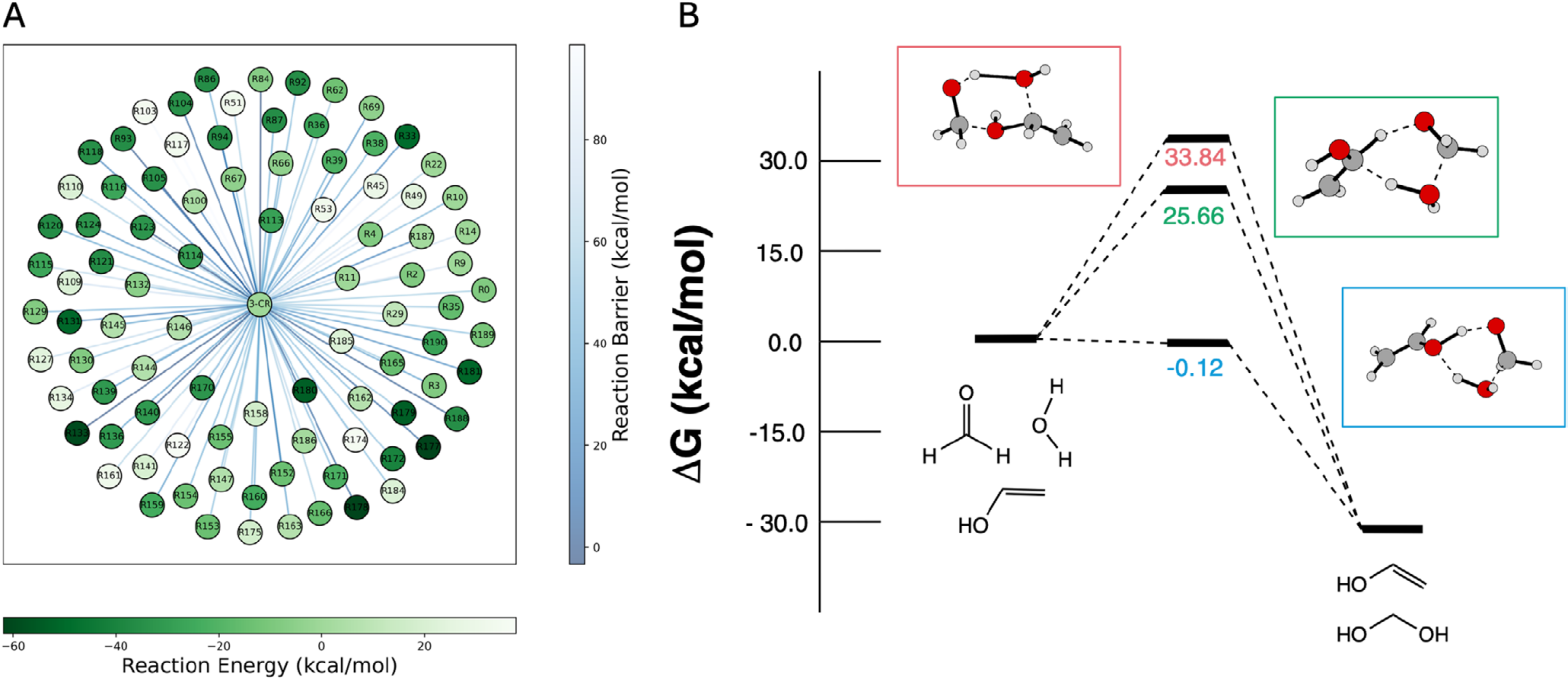
(A) Reaction networks of formaldehyde, water and vinyl alcohol depicting products as node and individual activation barriers as the edge colors; more intense colors means is more favorable. (B) Overview of some realistic mechanisms leading to methanediol formation.

A notable feature of the algorithm is its ability to identify multiple, mechanistically distinct pathways connecting the same set of reactants and products. Figure 5B highlights three different pathways leading to the formation of methanediol. The two lowest‐energy routes (green and blue frames) are catalyzed by the vinyl alcohol molecule. The most favorable pathway proceeds through a six‐membered‐ring transition state involving the hydroxyl group of the enol, while the second‐lowest pathway involves the apolar hydrogen atom and the sp_2_ carbon. In contrast, the third pathway corresponds to direct nucleophilic attack of the water molecule at the electrophilic sp_2_ carbon, followed by cleavage of the C–O bond and a subsequent attack on formaldehyde to yield the final product. At the GFN2‐xTB level of theory, the energetic separation between these pathways amounts to approximately 34 kcal mol^–1^.

When all stereoisomers of the 68 retained products and their associated reaction channels are taken into account, the enumeration yields a total of 195 distinct pathways for this 14‐atom system. The resulting reaction network was subsequently explored using TS‐tools [29], and 90 of these pathways were successfully recovered at the GFN2‐xTB level of theory (Figure 5A). The most thermodynamically favorable products are the two stereoisomers of 1‐(hydroxymethoxy)ethan‐1‐ol, characterized by reaction free energies of $\Delta_{r}G\approx -61$ kcal mol^–1^ and activation free energies of $\Delta G^{‡}\approx 5$ kcal mol^–1^. In addition, several aldol‐type products are identified, including 3‐hydroxypropanal, (vinyloxy)methanol, and methanediol, each of which can be accessed via multiple, distinct reaction pathways.

### 3.4 Generating Mechanistic Hypotheses for Organometallic Reactions

Enumeration algorithms frequently exclude TMCs because conventional graph representations can become ambiguous in these systems, yielding multiple competing topologies or even disconnected nodes. By constructing molecular graphs with the aid of NBO analysis, a chemically reasonable and internally consistent bonding description can instead be recovered, enabling the enumeration of realistic reaction pathways in organometallic systems.

**FIGURE 6:**
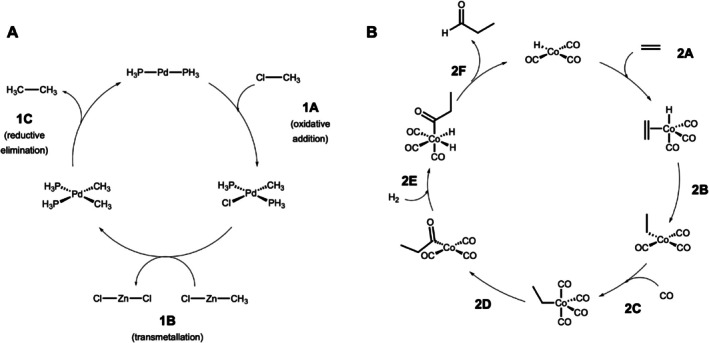
(A) Catalytic cycle of a prototypical Negishi coupling reaction. (B) Catalytic cycle for the Co‐catalyzed hydroformylation reaction.

To illustrate this capability, we investigate two prototypical catalytic cycles: a palladium‐catalyzed Negishi cross‐coupling reaction and a cobalt‐catalyzed hydroformylation reaction (Figure 6) [46, 47]. In both cases, the incorporation of NBO‐derived bonding information allows the full catalytic cycle to be reconstructed from elementary reaction steps, despite the presence of nonclassical bonding motifs and pronounced electron delocalization.

For the Negishi coupling reaction, the threshold for strong secondary interactions was adjusted to 50.0 kcal mol^–1^, which enabled recovery of the full catalytic cycle in an internally consistent manner. The thresholds for secondary interactions and the maximum intra‐fragment path length were otherwise left unchanged. Interestingly, a plausible alternative catalytic cycle was also enumerated (see Section S6 of the ESI). While this mechanism is unlikely to actually transpire due to an unfavorable energetic span, this example illustrates how the algorithm can, in principle, assist in the exploration and discovery of novel hypotheses for catalytic cycles.

In the case of the hydroformylation reaction (Figure 6B), the thresholds for secondary interactions had to be finetuned as well—the one for “strong” secondary interactions was set to 35.0 kcal/mol, and the threshold for regular secondary interactions was set to 6.0 kcal/mol. The maximum intra‐fragment path length was kept at its default value. With these settings, all intermediates from the commonly accepted catalytic cycle were readily recovered without further tinkering. Table 1 summarizes the number of enumerated products per step as well as the reaction energy of the interested product.

**TABLE 1:** Table summarizing the reaction steps depicted in Figure 6. For each step, the number of enumerated products and the corresponding reaction energies are reported.

Reaction summary					
Pd–Negishi coupling			Co‐catalyzed hydroformylation		
Step	Products	ΔE	Step	Products	ΔE
1A	11	−5.58	2A	5	−8.39
1B	51	4.94	2B	80	−5.51
1C	49	−32.50	2C	12	−13.47
			2D	138	3.76
			2E	9	9.34
			2F	142	−9.71

### 3.5 Exploration of Radical Reaction Networks

**FIGURE 7:**
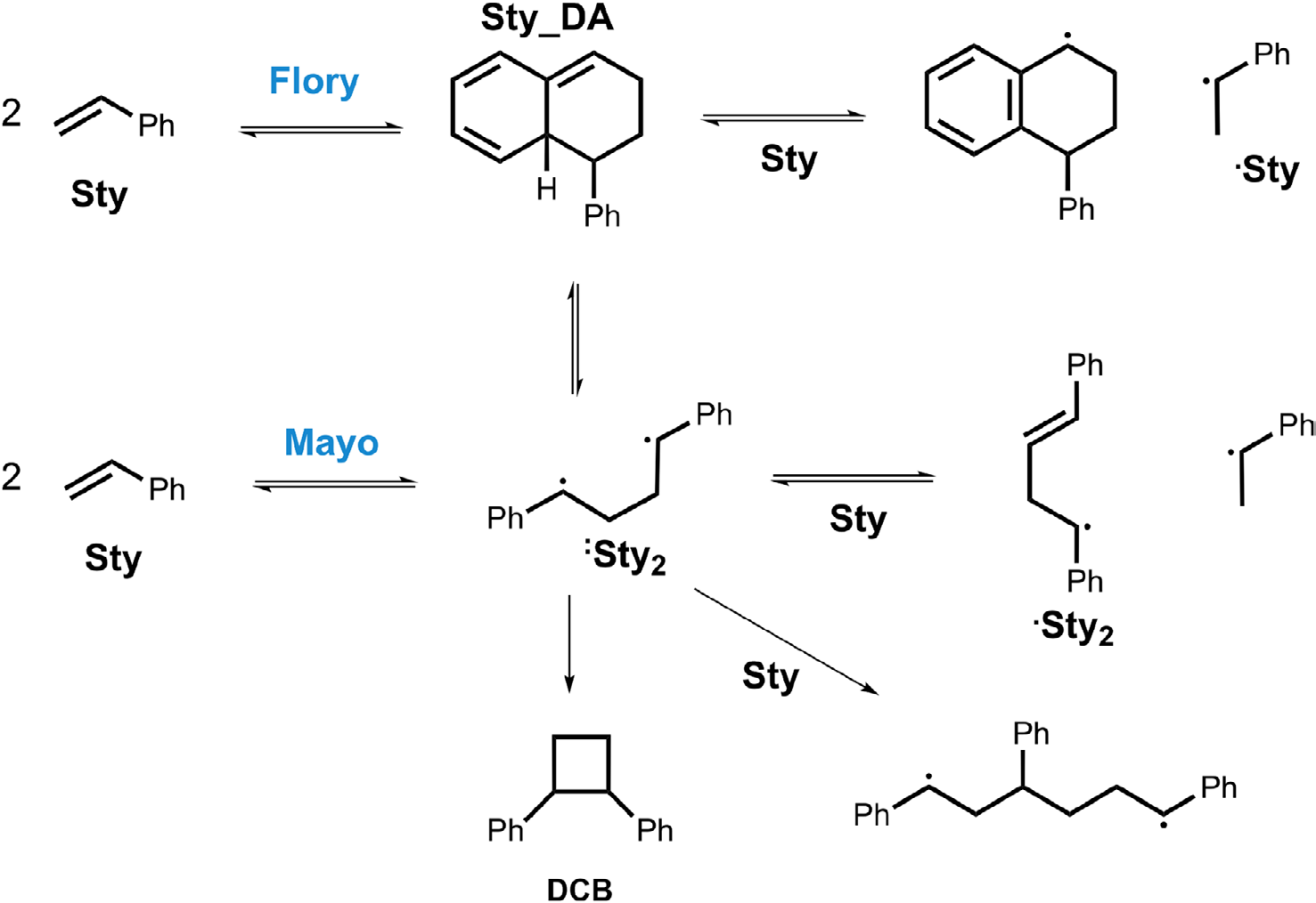
Mayo and Flory mechanisms.

The spontaneous thermal polymerization of styrene (**Sty**) has been investigated for nearly a century [48, 49, 50]. The most widely accepted mechanism is that proposed by Flory [49], in which two styrene molecules dimerize to form a singlet 1,4‐diradical (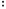 **Sty** 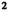). In the presence of a third styrene molecule, this diradical generates monoradical initiators that subsequently trigger the chain‐growth polymerization process. Alternatively, the diradical may undergo intramolecular cyclization to produce 1,2‐diphenylcyclobutane (**DCB**). An alternative mechanistic proposal, proposed by Mayo and co‐workers [50], involves an initial Diels–Alder dimerization of **Sty**. The resulting adduct (**Sty_DA**) can then undergo a molecule‐assisted homolysis with a third **Sty**, yielding monoradical initiators 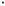 **Sty_DA** and 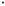 **Sty** that initiate polymer growth. These two mechanistic frameworks are summarized in Figure 7.

Our exploration here begins from the 1,4‐diradical 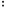 **Sty** 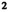. In a first enumeration step, a total of 1347 reaction paths were generated, of which 633 exhibit $\Delta_{r}E$ values below 50 kcal mol^–1^. All three major products associated with the established mechanistic proposals were recovered at this stage. Among them, **DCB** is identified as the most thermodynamically stable product ($\Delta_{r}E=$ −62.67 kcal mol^–1^), followed by the Diels–Alder adduct **Sty_DA** ($\Delta_{r}E=$ −52.56 kcal mol^–1^). Homolytic cleavage of the diradical back into two **Sty** molecules is also accessible, with $\Delta_{r}E=$ −17.35 kcal mol^–1^.

In a second round of exploration, 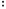 **Sty** 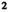 was allowed to react with an additional **Sty** molecule. This expanded network comprises 430 distinct products connected by 1014 reaction pathways. Importantly, both radical‐initiated polymerization routes ‐corresponding to the Flory and Mayo mechanisms‐ are recovered within this extended reaction space, demonstrating the ability of the enumeration protocol to capture competing initiation pathways within a unified automated mechanistic framework.

### 3.6 Unimolecular Degradation Network of γ‐ketohydroperoxide

As a final example, we investigated the unimolecular degradation of 3‐ketohydroperoxide (KHP) [51]. This system has previously been examined using graph‐based enumeration approaches up to the b4f4 level [23], yielding a total of 98 distinct products. As discussed above, transformations involving multiple simultaneous bond‐breaking and bond‐forming events are typically associated with prohibitively high kinetic barriers, except in cases where electronic conjugation stabilizes concerted rearrangements. For this reason, the default computational settings of our approach were retained for the present study.

Application of our enumeration protocol to KHP resulted in 68 thermodynamically feasible reaction pathways, of which 33 corresponding transition states were successfully validated (cf. Section S3.1 of the ESI for further details). At the GFN2‐xTB level of theory, the lowest activation barrier corresponds to the formation of 1,2‐dioxolan‐3‐ol (P1) via the Korcek mechanism, in agreement with previous experimental and computational studies [22, 51]. A second relatively low‐barrier pathway ($\Delta G^{‡}\approx 52$ kcal mol^–1^) leads to malonaldehyde (P3). In addition, our enumeration identifies 2‐hydroperoxycyclopropan‐1‐one (P2) as a previously unreported product of KHP degradation.

A second comparatively low‐barrier pathway ($\Delta G^{‡}\approx 52$ kcal mol^–1^) leads to the formation of malonaldehyde (P3). In addition, our enumeration protocol identifies 2‐hydroperoxycyclopropan‐1‐one (P2) as a previously unreported product of KHP degradation.

**FIGURE 8:**
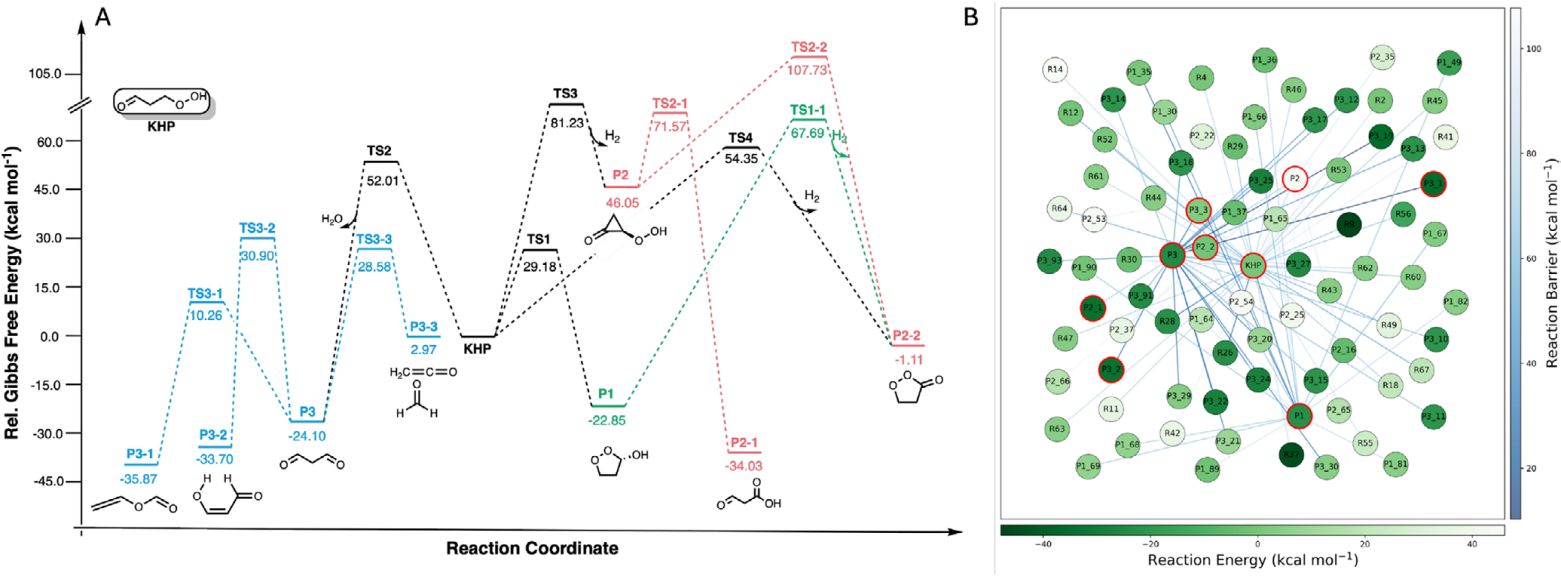
(A) Explored pathways for the uni‐molecular decomposition, in green the most kinetically favorable route, in blue the malondialdehyde channel and in red a newly discovery route. Energies are computed at GFN2‐xTB level of theory. Different line colors represent different starting points (i.e., reactants and intermediates). (B) Reaction networks of 3‐ketohydroperoxide (KHP) depicting products as node and individual activation barriers as the edge colors; more intense colors means is more favorable.

These three products‐P1, P2, and P3‐were subsequently treated as individual reactants in a second round of enumeration. As shown in Figure 8, KHP itself is not the most thermodynamically stable species within the resulting reaction network. Intramolecular rearrangements constitute the kinetically most accessible degradation pathways, as exemplified by formation of P1, whereas elimination of H_2_ or H_2_O incurs a substantially higher energetic penalty.

In addition, a competitive pathway leading to P3‐1 was identified. Although the precursor P3 is thermodynamically more stable than P1, it is accessed via a higher activation barrier. Once formed, however, P3 can undergo a subsequent facile intramolecular rearrangement to yield P3‐1, which emerges as the most thermodynamically stable product identified in the network, and may thus be experimentally observable when the reaction is run at sufficiently high temperatures. Finally, P2 was explored in detail precisely because it had not been reported in previous enumeration studies, illustrating the ability of the present approach to uncover novel degradation channels beyond established reaction hypotheses.

## 4 Conclusions

In this work, we have presented a novel graph‐based reaction‐pathway enumeration framework in which molecular graphs are formulated at the level of VOs, and reaction products are generated through orbital rearrangement and/or redistribution of the electrons residing in them. By coupling this representation with NBO analysis, the method can explicitly encode electronic delocalization, multicenter bonding, and stereoelectronic effects, features that are particularly important for reaction classes that challenge conventional graph‐based approaches.

We demonstrate the capabilities of the proposed algorithm across several challenging reaction classes, including organometallic and multicomponent transformations that often lie beyond the scope of conventional graph‐based enumeration protocols. Pericyclic reactions constitute a particular class of transformations for which the orbital‐graph framework proves especially well suited. In this context, the explicit inclusion of secondary interactions enables the algorithm to mitigate the otherwise prohibitive combinatorial growth in enumerated products, while still recovering the chemically relevant cycloaddition products. Finally, validation on unimolecular reaction networks and bimolecular benchmark sets shows that our protocol achieves performance comparable to established enumeration approaches in its ability to identify chemically meaningful transformations.

It is well established that NBO analyses are inherently dependent on the three‐dimensional molecular structure, and this dependence is reflected in the stereoelectronic effects observed in flexible systems. In cases where multiple competing conformational minima exist, incomplete conformational sampling may therefore lead to the omission of some secondary interactions. In the present work, this sensitivity has been partially mitigated, by applying an electronic cut‐off that filters weak, highly conformer‐dependent interactions that are unlikely to play a decisive role in chemical reactivity.

Within these bounds, the proposed orbital‐graph framework enables a qualitatively new balance between chemical realism and combinatorial control, allowing electronically complex reaction classes to be explored systematically without sacrificing tractability. As such, the current work establishes a flexible platform for future extensions that incorporate enhanced conformational sampling or predictive electronic‐structure surrogates, further broadening the applicability of graph‐based reaction discovery.

## Funding

This work was supported by the Agence Nationale de la Recherche (Grant Nos. ANR‐22‐CPJ1‐0093‐01 and ANR‐24‐CE29‐5745) and the Grand Équipement National De Calcul Intensif (Grant No. 2023‐100732).

## Conflicts of Interest

The authors declare no conflicts of interest.

## Supporting information




**Section S1:** Valence orbital graph procedure.
**Section S2:** Sensitivity analysis of the secondary interactions towards the selected level‐of‐theory.
**Figure S1:** Examples cases.
**Table S1:** Sensitivity of the SOPA stabilization energies (kcal mol^–1^) to the level of theory for the donor–acceptor interactions in the Pd complex (Figure S1A).
**Section S3:** TS‐tools methodology.
**Table S2:** Sensitivity of the SOPA stabilization energies (kcal mol^–1^) to the level of theory for the donor–acceptor interactions in the azide dipole (Figure S1B).
**Section S3.1:** Main sources of failure of transition state searches.
**Section S4:** Sensitivity analysis towards the threshold values.
**Table S3:** Number of products obtained for the pericyclic set based on secondary interactions threshold.
**Table S4:** Number of products obtained for the benchmarking set based on secondary interactions threshold.
**Table S5:** Number of products obtained for the Negishi coupling reaction based on strong secondary interactions threshold.
**Section S5:** Calibration guidelines for threshold‐sec‐int and threshold‐strong‐sec‐int.
**Section S6:** Hypothetical alternative Negishi coupling reaction.
**Section S7:** Cleaned up version of reaction benchmarking dataset.
**Table S6:** Table summarizing the reaction steps depicted in Figure S2. For each step, the number of enumerated products and the corresponding reaction energies are reported.
**Figure S2:** Alternative catalytic cycle of a Negishi coupling reaction.
**Table S7:** Results for the 85 reactions curated as part of our study. A value of YES indicates that the corresponding product is successfully enumerated, whereas NO indicates that the product is not enumerated.

## Data Availability

The enumeration procedure is implemented in a Python package and can be found at https://github.com/chimie‐paristech‐CTM/reaction_possibility_enumerator. Raw data sources generated by this work are available at https://figshare.com/projects/Enumerator/271312. The data that supports the findings of this study are available in the Supporting Information of this article.

## References

[1] I. Ugi , J. Bauer , J. Brandt , et al., “New Applications of Computers in Chemistry,” Angewandte Chemie International Edition in English 18, no. 2 (1979): 111–123.

[2] J. Dugundji and I. Ugi , “An Algebraic Model of Constitutional Chemistry as a Basis for Chemical Computer Programs,” in Computers in Chemistry (Springer Berlin Heidelberg, 1973), 19–64.

[3] J. P. Unsleber and M. Reiher , “The Exploration of Chemical Reaction Networks,” Annual Review of Physical Chemistry 71, no. 1 (2020): 121–142. 10.1146/annurev-physchem-071119-040123 32105566

[4] Z. Tu , T. Stuyver , and C. W. Coley , “Predictive Chemistry: Machine Learning for Reaction Deployment, Reaction Development, and Reaction Discovery,” Chemical Science 14, no. 2 (2023): 226–244. 36743887 10.1039/d2sc05089g PMC9811563

[5] S. Maeda and K. Morokuma , “Finding Reaction Pathways of Type a + b → x: Toward Systematic Prediction of Reaction Mechanisms,” Journal of Chemical Theory and Computation 7, no. 8 (2011): 2335–2345. 26606607 10.1021/ct200290m

[6] S. Maeda and K. Morokuma , “Toward Predicting Full Catalytic Cycle Using Automatic Reaction Path Search Method: A Case Study on HCo(CO)_3_‐Catalyzed Hydroformylation,” Journal of Chemical Theory and Computation 8, no. 2 (2012): 380–385. 26596590 10.1021/ct200829p

[7] L.‐P. Wang , A. Titov , R. McGibbon , F. Liu , V. S. Pande , and T. J. Martínez , “Discovering Chemistry With an Ab Initio Nanoreactor,” Nature Chemistry 6 (2014): 1044–1048. 10.1038/nchem.2099 PMC4239668 25411881

[8] E. Pieri , D. Lahana , A. M. Chang , C. R. Aldaz , K. C. Thompson , and T. J. Martínez , “The Non‐Adiabatic Nanoreactor: Towards the Automated Discovery of Photochemistry,” Chemical Science 12 (2021): 7294–7307. 34163820 10.1039/d1sc00775k PMC8171323

[9] J. Ford , S. Seritan , X. Zhu , et al., “Nitromethane Decomposition via Automated Reaction Discovery and an Ab Initio Corrected Kinetic Model,” Journal of Physical Chemistry A 125, no. 7 (2021): 1447–1460. 33569957 10.1021/acs.jpca.0c09168

[10] G. N. Simm and M. Reiher , “Context‐Driven Exploration of Complex Chemical Reaction Networks,” Journal of Chemical Theory and Computation 13, no. 12 (2017): 6108–6119. 29084387 10.1021/acs.jctc.7b00945

[11] J. P. Unsleber , S. A. Grimmel , and M. Reiher , “Chemoton 2.0: Autonomous Exploration of Chemical Reaction Networks,” Journal of Chemical Theory and Computation 18, no. 9 (2022): 5393–5409. 35926118 10.1021/acs.jctc.2c00193 PMC11516015

[12] M. Bensberg , S. Grimmel , L. Lang , et al., “qcscine/chemoton: Release 4.1.0,” 2025.

[13] S. Habershon , “Sampling Reactive Pathways With Random Walks in Chemical Space: Applications to Molecular Dissociation and Catalysis,” Journal of Chemical Physics 143, no. 9 (2015): 094106. 26342358 10.1063/1.4929992

[14] S. Habershon , “Automated Prediction of Catalytic Mechanism and Rate Law Using Graph‐Based Reaction Path Sampling,” Journal of Chemical Theory and Computation 12, no. 4 (2016): 1786–1798. 26938837 10.1021/acs.jctc.6b00005

[15] M. Bergeler , G. N. Simm , J. Proppe , and M. Reiher , “Heuristics‐Guided Exploration of Reaction Mechanisms,” Journal of Chemical Theory and Computation 11, no. 12 (2015): 5712–5722. 26642988 10.1021/acs.jctc.5b00866

[16] C. W. Gao , J. W. Allen , W. H. Green , and R. H. West , “Reaction Mechanism Generator: Automatic Construction of Chemical Kinetic Mechanisms,” Computer Physics Communications 203 (2016): 212–225.

[17] J. F. Joung , M. H. Fong , N. Casetti , J. P. Liles , N. S. Dassanayake , and C. W. Coley , “Electron Flow Matching for Generative Reaction Mechanism Prediction,” Nature 645 (2025): 115–123. 40836082 10.1038/s41586-025-09426-9

[18] P. M. Zimmerman , “Automated Discovery of Chemically Reasonable Elementary Reaction Steps,” Journal of Computational Chemistry 34, no. 16 (2013): 1385–1392. 23508333 10.1002/jcc.23271

[19] I. M. Pendleton , M. H. Pérez‐Temprano , M. S. Sanford , and P. M. Zimmerman , “Experimental and Computational Assessment of Reactivity and Mechanism in C(sp3)‐N Bond‐Forming Reductive Elimination From Palladium(IV),” Journal of the American Chemical Society 138, no. 18 (2016): 6049–6060. 27087364 10.1021/jacs.6b02714

[20] P. M. Zimmerman , “Single‐Ended Transition State Finding With the Growing String Method,” Journal of Computational Chemistry 36, no. 9 (2015): 601–611. 25581279 10.1002/jcc.23833

[21] M. Woulfe and B. M. Savoie , “Chemical Reaction Networks From Scratch With Reaction Prediction and Kinetics‐Guided Exploration,” Journal of Chemical Theory and Computation 21, no. 3 (2025): 1276–1291. 39883589 10.1021/acs.jctc.4c01401

[22] Q. Zhao and B. M. Savoie , “Algorithmic Explorations of Unimolecular and Bimolecular Reaction Spaces,” Angewandte Chemie (International Edition in English) 61, no. 46 (2022): e202210693. 10.1002/anie.202210693 PMC9827825 36074520

[23] Q. Zhao and B. M. Savoie , “Simultaneously Improving Reaction Coverage and Computational Cost in Automated Reaction Prediction Tasks,” Nature Computational Science 1 (2021): 479–490. 38217124 10.1038/s43588-021-00101-3

[24] N. Casetti , D. Anstine , O. Isayev , and C. W. Coley , “Anticipating the Selectivity of Intramolecular Cyclization Reaction Pathways With Neural Network Potentials,” Journal of Chemical Theory and Computation 21, no. 20 (2025): 10362–10372. 41066459 10.1021/acs.jctc.5c01161

[25] E. D. Glendening , C. R. Landis , and F. Weinhold , “Natural Bond Orbital Methods,” WIREs Computational Molecular Science 2, no. 1 (2012): 1–42.

[26] H. Kneiding , R. Lukin , L. Lang , et al., “Deep Learning Metal Complex Properties With Natural Quantum Graphs,” Digital Discovery 2 (2023): 618–633.

[27] H. Kneiding , A. Nova , and D. Balcells , “Directional Multiobjective Optimization of Metal Complexes at the Billion‐System Scale,” Nature Computational Science 4 (2024): 263–273. 38553635 10.1038/s43588-024-00616-5

[28] P. Zimmerman , “Reliable Transition State Searches Integrated With the Growing String Method,” Journal of Chemical Theory and Computation 9, no. 7 (2013): 3043–3050. 26583985 10.1021/ct400319w

[29] T. Stuyver , “TS‐Tools: Rapid and Automated Localization of Transition States Based on a Textual Reaction Smiles Input,” Journal of Computational Chemistry 45, no. 27 (2024): 2308–2317. 38850166 10.1002/jcc.27374

[30] D. A. Boiko , T. Reschützegger , B. Sanchez‐Lengeling , S. M. Blau , and G. Gomes , “Advancing Molecular Machine Learning Representations With Stereoelectronics‐Infused Molecular Graphs,” Nature Machine Intelligence 7 (2025): 771–781.

[31] G. C. Pimentel , “The Bonding of Trihalide and Bifluoride Ions by the Molecular Orbital Method,” Journal of Chemical Physics 19, no. 4 (1951): 446–448.

[32] R. J. Gillespie and B. Silvi , “The Octet Rule and Hypervalence: Two Misunderstood Concepts,” Coordination Chemistry Reviews 233–234 (2002): 53–62.

[33] B. Braïda and P. C. Hiberty , “The Essential Role of Charge‐Shift Bonding in Hypervalent Prototype XeF_2_ ,” Nature Chemistry 5 (2013): 417–422. 10.1038/nchem.1619 23609093

[34] D. Weininger , “SMILES, A Chemical Language and Information System. 1. Introduction to Methodology and Encoding Rules,” Journal of Chemical Information and Computer Sciences 28, no. 1 (1988): 31–36.

[35] D. Weininger , A. Weininger , and J. L. Weininger , “SMILES. 2. Algorithm for Generation of Unique SMILES Notation,” Journal of Chemical Information and Computer Sciences 29, no. 2 (1989): 97–101.

[36] T. A. Young , J. J. Silcock , A. J. Sterling , and F. Duarte , “autodE: Automated Calculation of Reaction Energy Profiles‐Application to Organic and Organometallic Reactions,” Angewandte Chemie International Edition 133, no. 8 (2021): 4312–4320. 10.1002/anie.202011941 33108028

[37] C. Bannwarth , S. Ehlert , and S. Grimme , “GFN2‐xTB‐An Accurate and Broadly Parametrized Self‐Consistent Tight‐Binding Quantum Chemical Method With Multipole Electrostatics and Density‐Dependent Dispersion Contributions,” Journal of Chemical Theory and Computation 15, no. 3 (2019): 1652–1671. 30741547 10.1021/acs.jctc.8b01176

[38] M. J. Frisch , G. W. Trucks , H. B. Schlegel , et al., “Gaussian 16,” 2016.

[39] E. D. Glendening , J. K. Badenhoop , A. E. Reed , et al., “NBO 7. 0,” 2018.

[40] C. Adamo and V. Barone , “Toward Reliable Density Functional Methods Without Adjustable Parameters: The PBE0 Model,” Journal of Chemical Physics 110, no. 13 (1999): 6158–6170.

[41] A. Schäfer , H. Horn , and R. Ahlrichs , “Fully Optimized Contracted Gaussian Basis Sets for Atoms Li to Kr,” Journal of Chemical Physics 97, no. 4 (1992): 2571–2577.

[42] RDKit , “Open‐Source Cheminformatics”.

[43] T. Stuyver , K. Jorner , and C. W. Coley , “Reaction Profiles for Quantum Chemistry‐Computed [3+2] Cycloaddition Reactions,” Scientific Data 10, no. 1 (2023): 66. 36725850 10.1038/s41597-023-01977-8 PMC9892576

[44] T. Stuyver and C. W. Coley , “Machine Learning‐Guided Computational Screening of New Candidate Reactions With High Bioorthogonal Click Potential,” Chemistry—A European Journal 29 (2023): e202300387. 36787246 10.1002/chem.202300387

[45] X.‐S. Xue , C. S. Jamieson , M. Garcia‐Borràs , X. Dong , Z. Yang , and K. N. Houk , “Ambimodal Trispericyclic Transition State and Dynamic Control of Periselectivity,” Journal of the American Chemical Society 141, no. 3 (2019): 1217–1221. 30623652 10.1021/jacs.8b12674

[46] V. B. Phapale and D. J. Crdenas , “Nickel‐Catalysed Negishi Cross‐Coupling Reactions: Scope and Mechanisms,” Chemical Society Reviews 38 (2009): 1598–1607. 19587955 10.1039/b805648j

[47] R. F. Heck and D. S. Breslow , “The Reaction of Cobalt Hydrotetracarbonyl With Olefins,” Journal of the American Chemical Society 83, no. 19 (1961): 4023–4027.

[48] K. S. Khuong , W. H. Jones , W. A. Pryor , and K. N. Houk , “The Mechanism of the Self‐Initiated Thermal Polymerization of Styrene. Theoretical Solution of a Classic Problem,” Journal of the American Chemical Society 127, no. 4 (2005): 1265–1277. 15669866 10.1021/ja0448667

[49] P. J. Flory , “The Mechanism of Vinyl Polymerizations,” Journal of the American Chemical Society 59, no. 2 (1937): 241–253.

[50] F. R. Mayo , “Chain Transfer in the Polymerization of Styrene. Viii. Chain Transfer With Bromobenzene and Mechanism of Thermal Initiations,” Journal of the American Chemical Society 75, no. 24 (1953): 6133–6141.

[51] A. Jalan , I. M. Alecu , R. Meana‐Pañeda , et al., “New Pathways for Formation of Acids and Carbonyl Products in Low‐Temperature Oxidation: The Korcek Decomposition of γ‐Ketohydroperoxides,” Journal of the American Chemical Society 135, no. 30 (2013): 11100–11114. 23862563 10.1021/ja4034439

