# Supplementary material for: Reaction Enumeration Based on NBO‐Informed Molecular Graphs
Source: J Comput Chem. 2026 Jul 6;47(19):e70462. doi: 10.1002/jcc.70462 (PMC13338458; doi:10.1002/jcc.70462)
Supplement: Supplementary file 1 — Section S1: Valence orbital graph procedure. Section S2: Sensitivity analysis of the secondary interactions towards the selected level‐of‐theory. Figure S1: Examples cases. Table S1: Sensitivity of the SOPA stabilization energies (kcal mol–1) to the level of theory for the donor–acceptor interactions in the Pd complex (Figure S1A). Section S3: TS‐tools methodology. Table S2: Sensitivity of the SOPA stabilization energies (kcal mol–1) to the level of theory for the donor–acceptor interactions in the azide dipole (Figure S1B). Section S3.1: Main sources of failure of transition state searches. Section S4: Sensitivity analysis towards the threshold values. Table S3: Number of products obtained for the pericyclic set based on secondary interactions threshold. Table S4: Number of products obtained for the benchmarking set based on secondary interactions threshold. Table S5: Number of products obtained for the Negishi coupling reaction based on strong secondary interactions threshold. Section S5: Calibration guidelines for threshold‐sec‐int and threshold‐strong‐sec‐int. Section S6: Hypothetical alternative Negishi coupling reaction. Section S7: Cleaned up version of reaction benchmarking dataset. Table S6: Table summarizing the reaction steps depicted in Figure S2. For each step, the number of enumerated products and the corresponding reaction energies are reported. Figure S2: Alternative catalytic cycle of a Negishi coupling reaction. Table S7: Results for the 85 reactions curated as part of our study. A value of YES indicates that the corresponding product is successfully enumerated, whereas NO indicates that the product is not enumerated. [file JCC-47-0-s001.pdf]

Supporting Information
to
Reaction Enumeration Based on NBO-informed Molecular Graphs

Javier E. Alfonso-Ramos and Thijs Stuyver*

Ecole Nationale Supérieure de Chimie de Paris, Université PSL, CNRS, i-CLeHS, 75 005 Paris, France

E-mail: thijs.stuyver@chimieparistech.psl.eu

Contents

S1 Valence orbital graph procedure **S3**

S2 Sensitivity analysis of the secondary interactions towards the selected level-of-theory **S3**

S3 TS-Tools methodology **S4**

S3.1 Main sources of failure of transition state searches S7

S4 Sensitivity analysis towards the threshold values **S7**

S5 Calibration guidelines for threshold-sec-int and threshold-strong-sec-int **S9**

S6 Hypothetical alternative Negishi coupling reaction **S10**

S7 Cleaned up version of reaction benchmarking dataset **S10**

References **S12**

S1 Valence orbital graph procedure

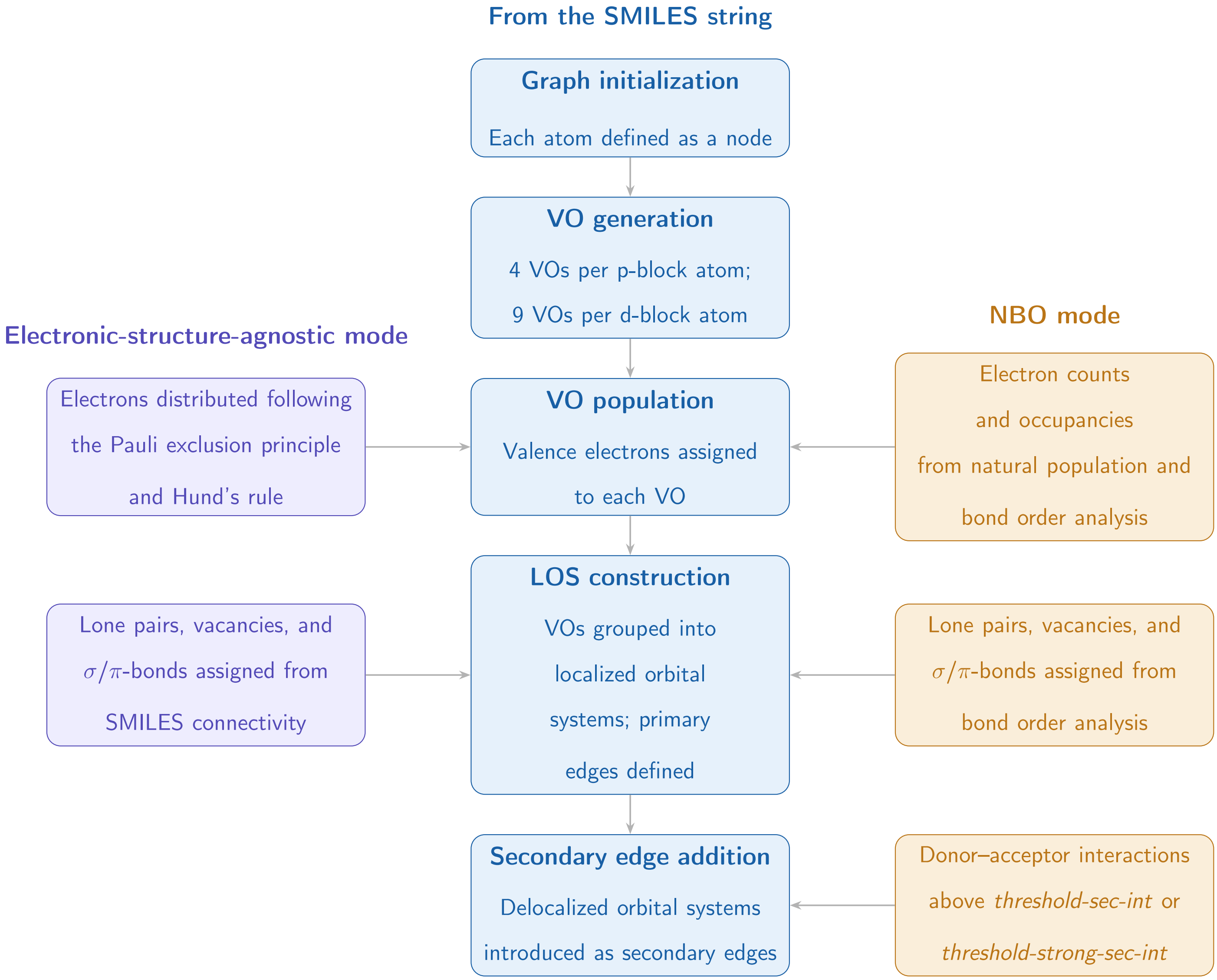

S2 Sensitivity analysis of the secondary interactions towards the selected level-of-theory

Four functionals (B97D3, PBE1PBE, M06-2X, and ωB97XD) in combination with two basis sets (def2-SVP and def2-TZVP) were tested. As the number of products typically did not increase significantly upon tuning the threshold for secondary interactions, we focused on two

representative examples. The first assesses the ability to recover the full bonding pattern of the Pd complex, while the second highlights a relevant secondary interaction, namely π-delocalization in the 1,3-dipole.

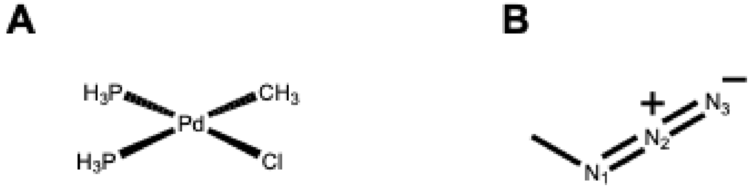

Figure S1: Examples cases

Table S1: Sensitivity of the SOPA stabilization energies (kcal mol^{-1}) to the level of theory for the donor–acceptor interactions in the Pd complex (Fig. S1A).

Interaction	LP (P_1) – BD (C - Pd)	
Functional \ Basis Set	def2-SVP	def2-TZVP
ωB97XD	139.6	141.8
M062X	124.9	117.0
PBE1PBE	116.7	124.0
B97XD3	94.4	95.2
Interaction	LP (Cl) – BD (P_2 - Pd)	
ωB97XD	120.3	134.0
M062X	103.8	116.9
PBE1PBE	93.6	107.4
B97XD3	75.7	87.4

S3 TS-Tools methodology

TS-tools is an open-source Python package,[1] originally developed for the automated localization of transition states (TSs) starting from textual (atom-mapped) reaction SMILES inputs.[2,3] One of its main strengths is the ability to identify TS geometries for tri- and multimolecular reaction pathways, which are relevant for many types of reactivity, such as

Table S2: Sensitivity of the SOPA stabilization energies (kcal mol^{-1}) to the level of theory for the donor–acceptor interactions in the azide dipole (Fig. S1B).

Interaction	LP (N_1) – BD (N_2 - N_3)	
Functional \ Basis Set	def2-SVP	def2-TZVP
ωB97XD	34.9 (1)	31.6 (4)
M062X	29.4 (3)	26.6 (4)
PBE1PBE	21.6 (2)	19.4 (4)
B97XD3	13.7 (1)	12.0 (1)

solvent- and autocatalysis as well as enzymatic reactions. In its current implementation, the TS-tools codebase is structured around two core Python classes: PathGenerator and TSOptimizer. The overall workflow is described in detail below.

The first step consists of generating an initial reactive complex using the PathGenerator class. Reactant and product SMILES strings are compared, and so-called active bonds are identified with the aid of RDKit. Active bonds are defined as bonds that change during the course of the reaction. Optimal bond lengths are determined for each active bond, and an initial conformer is generated using autodE's *randomize-and-relax* force-field procedure.[4] During this step, the bonds being formed are constrained to ideal product bond lengths multiplied by a stretching parameter. In this way, the reactants are pre-organized to facilitate a smooth reaction pathway from reactants to products.

An important verification step is performed at this stage: the stereochemistry of the generated reactive complex must match that specified in the reactant SMILES. If this condition is not satisfied, the procedure is repeated until the correct stereochemistry is obtained or until a maximum of 100 unsuccessful iterations is reached. The resulting reactive complex is subsequently optimized at the xTB level of theory while maintaining the same constraints on the forming bonds.

Next, an Artificial Force Induced Reaction (AFIR)[5] pathway is generated through a sequence of pull and push steps that apply external forces to overcome the reaction barrier.

Specifically, a biased optimization is performed on the reactant complex geometry, in which constraining potentials are applied to the forming bonds with target distances corresponding to the ideal product bond lengths. To determine the minimum force required to drive the system from reactants to products, the force constant is increased in increments of 0.1 au (Hartree Bohr^{-2}). At each step, the total external potential is compared to a predefined threshold. Once this threshold is reached, a more refined search is carried out using smaller force increments. Finally, the connectivity of the optimized structure is compared with that of the product to verify the success of the reactive pathway. If a successful path is identified, the intermediate geometries and their associated energies are stored for further analysis.

The TSOptimizer class begins by generating a reactive pathway for a given stretching factor using the PathGenerator. Local maxima along this pathway are selected as initial TS guesses. A first filtering step is then applied to remove structures that are unlikely to correspond to true transition states. In particular, structures are discarded if they exhibit an imaginary frequency below 150 cm^{-1} and/or if the corresponding imaginary mode does not primarily involve bond formation or cleavage. The remaining candidates are ranked by energy, and the five highest-energy structures are selected as inputs for transition-state optimization.

Transition-state optimizations are performed at the xTB level of theory using Gaussian16, interfaced through a Python wrapper.[6] Each TS optimization is followed by an intrinsic reaction coordinate (IRC) calculation to confirm that the optimized TS connects the correct reactant and product states. The connectivities of the IRC endpoints are compared to those of the corresponding start and end points of the reactive path; if they match, the TS is considered validated. Further refinement at the density functional theory (DFT) level is possible, and in some cases necessary, either by reoptimizing the TS or by performing the TS optimization directly at the DFT level to ensure successful localization.

In its current state, TS-tools is capable of generating a complete reaction profile. With the aid of CREST,[7] constrained conformational sampling of transition states is performed,

followed by validation using the TSOptimizer class to ensure that the correct imaginary vibrational mode is preserved throughout the conformational search. For reactants and products, conformational searches are carried out on the separated species derived from the respective complexes, ensuring stereochemical compatibility between all stationary points along the reaction pathway.

S3.1 Main sources of failure of transition state searches

As explained in the previous section, TS-Tools begins by generating an initial reactive complex in which the bonds being formed are constrained to specific bond lengths. These bond lengths are obtained by multiplying the ideal product bond length by a stretching parameter. If the first trial fails, additional stretching parameters are tested; by default, up to four stretching parameters are employed. During the generation of the reactive pathway, several structures are selected as initial transition-state (TS) guesses and subsequently optimized until a TS is located and validated. Because the protocol is repeated multiple times before concluding that a TS cannot be found, failures cannot always be attributed to a single source. For both the trimolecular reaction network and the unimolecular degradation network of γ-ketohydroperoxide, the most common causes of failure were the inability to generate a reactive complex consistent with the stereochemistry of the products and failures during the TS optimization step.

S4 Sensitivity analysis towards the threshold values

Table S3 reports the number of products generated by our enumeration algorithm as a function of the *threshold-sec-int* parameter. Green entries indicate that all products have been fully enumerated, whereas red entries denote cases where some products are missing. The reported values correspond to the total number of products, with the numbers in parentheses indicating the number of distinct reaction pathways.

Table S3: Number of products obtained for the pericyclic set based on secondary interactions threshold.

Reaction	Threshold (kcal mol^{-1})				
	8.0	10.0	12.0	14.0	16.0
[4+2] Diels-Alder	94 (300)	94 (300)	94 (300)	94 (300)	97 (340)
Click (3+2) cycloaddition	82 (124)	76 (104)	76 (104)	68 (95)	68 (95)
Salvinorin A	753 (5370)	753 (5370)	795 (5699)	753 (5370)	717 (5040)
Tropane + fulvene	255 (3464	255 (3464)	255 (3464)	231 (3174)	214 (3003)

Table S4 reports the number of products recovered by our enumeration algorithm for the benchmarking set as a function of the secondary interaction threshold. The reported values correspond to the total number of recovered products, while the numbers in parentheses indicate the total number of enumerated products.

Table S4: Number of products obtained for the benchmarking set based on secondary interactions threshold.

Benchmarking set	Threshold (kcal mol^{-1})				
	8.0	10.0	12.0	14.0	16.0
Total of 85 products	62 (758)	62 (755)	62 (755)	62 (751)	61 (749)

Table S5 reports the number of products generated by our enumeration algorithm as a function of the *threshold-strong-sec-int* parameter. Green entries indicate that the main product have been enumerated, whereas red entries denote cases where the main product is missing. The reported values correspond to the total number of products, with the numbers in parentheses indicating the number of distinct reaction pathways.

Table S5: Number of products obtained for the Negishi coupling reaction based on strong secondary interactions threshold.

Step	Threshold (kcal mol^{-1})				
	45.0	65.0	85.0	105.0	125.0
Oxidative Addition (1A)	11 (21)	11 (21)	11 (21)	11 (21)	11 (21)
Transmetallation (2A)	51 (121)	51 (121)	51 (121)	31 (93)	28 (95)
Reductive Elimination (3A)	49 (179)	49 (179)	49 (179)	46 (175)	45 (152)

S5 Calibration guidelines for threshold-sec-int and threshold-strong-sec-int

As explained in the main manuscript, the valence orbital graph framework relies on two energetic thresholds to filter donor–acceptor interactions identified in the second-order perturbation analysis (SOPA) of the NBO output. Here we provide practical guidance for their calibration.

1. Initial calibration from reactants

A practical starting point is to inspect the SOPA stabilization energies of the reactant structures, which typically already reflect the key bonding motifs of the system. In particular, the distribution of stabilization energies often exhibits a natural separation between weak interactions and strongly stabilizing donor–acceptor interactions (e.g., metal–ligand back-donation). These features can be used to define an initial choice of both *threshold-sec-int* and *threshold-strong-sec-int* before any enumeration is performed.

2. System dependence and metal-specific effects

The appropriate thresholds depend on the metal center, ligand environment, and overall electronic structure. This is especially true for *threshold-strong-sec-int*, which is sensitive to the nature of the metal and should therefore be calibrated on a per-system basis using the SOPA energy scale. Unlike *threshold-sec-int*, for which a default value of 12 kcal mol^{-1} provides a reasonable starting point across a broad range of main-group systems, no universal value for *threshold-strong-sec-int* can be recommended, and system-specific inspection of the SOPA output is essential.

3. Feedback-based refinement during enumeration

Rather than relying on trial-and-error, we recommend a feedback-based refinement strategy in which the SOPA analysis of key intermediates identified during the enumeration process is inspected iteratively. Two diagnostic signals guide this refinement:

- If chemically expected bonding motifs are absent in the intermediates (e.g., incomplete

coordination spheres around the metal center), the threshold is likely set too high and should be lowered.

- If excessive or chemically implausible delocalization is detected, the threshold is likely set too low and should be raised.

4. Exploration vs. robustness trade-off

The choice of threshold also reflects an inherent trade-off between enumeration breadth and robustness. Higher thresholds yield a more conservative enumeration, in which reactivity is assumed to be largely constrained to localized bonding systems, reducing combinatorial complexity and limiting the risk of spurious pathways. Lower thresholds enable broader exploration of alternative reaction mechanisms, including those involving non-classical or delocalized bonding motifs, at the cost of an increased number of candidate pathways that must be evaluated.

S6 Hypothetical alternative Negishi coupling reaction

The alternative mechanism identified using our enumeration protocol differs from the commonly reported a1B transmetallation pathway (*cf.* Fig. S2). In this mechanism, the organozinc reagent coordinates to the Pd(II) complex, while the methyl ligand on palladium couples with the methyl ligand on zinc to form ethane. In the subsequent step, the chloride ligand transfers to zinc, resulting in regeneration of the Pd(0) complex. Table S6 summarizes the number of enumerated products per step as well as the reaction energy of the interested product.

S7 Cleaned up version of reaction benchmarking dataset

From the original 105 benchmarking reactions curated by Zimmermann,[8,9] the 9 reactions determined to be not elementary[10] (R6, R10, R11, R20, R35, R54, R68, R90, R96) were

Table S6: Table summarizing the reaction steps depicted in Fig. S2. For each step, the number of enumerated products and the corresponding reaction energies are reported.

Reaction Summary		
Alternative Pd–Negishi		
step	products	ΔG
a1A	11	-5.58
a1B	51	-30.00
a1C	48	2.45

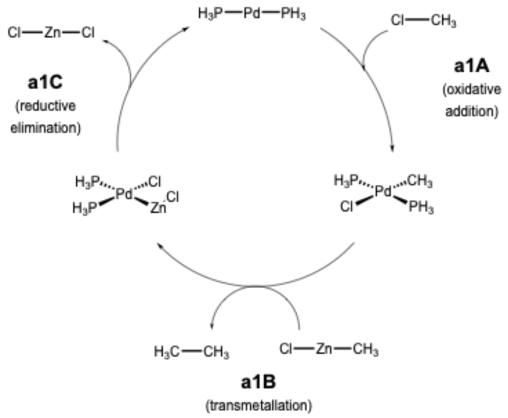

Figure S2: Alternative catalytic cycle of a Negishi coupling reaction

removed. Subsequently, in 3 reactions, the product can not be parsed by RDKit, and were consequently removed as well (R9, R15, R16). 6 reactions involved the same reaction SMILES (R19/R22, R83/R87, R92/R93), and hence the latter reaction was always removed. 2 reactions involved a conformational rearrangement (R94 and R95) and were discarded. 3 reactions involving the taxadiene carbocation were also removed. Finally, 6 reactions were determined to be intramolecular, but a "spectator" molecule was include in the reaction SMILES (R12, R48, R53, R70, R86, R99), those cases, the inactive molecules were removed from the reaction SMILES. A total of 20 reactions were removed. The final list of 83 reaction SMILES can be found in the Github repository.

Table S7 shows the results of applying our enumerator algorithms in the curated set of reactions.

References

(1) Stuyver, T. TS-tools: Rapid and automated localization of transition states based on a textual reaction SMILES input. *J. Comp. Chem.* **2024**, *45*, 2308–2317.

(2) Weininger, D. SMILES, a chemical language and information system. 1. Introduction to methodology and encoding rules. *J. Chem. Inf. Comput. Sci.* **1988**, *28*, 31–36.

(3) Weininger, D.; Weininger, A.; Weininger, J. L. SMILES. 2. Algorithm for generation of unique SMILES notation. *J. Chem. Inf. Comput. Sci.* **1989**, *29*, 97–101.

(4) Young, T. A.; Silcock, J. J.; Sterling, A. J.; Duarte, F. autodE: automated calculation of reaction energy profiles—application to organic and organometallic reactions. *Angew. Chem., Int. Ed.* **2021**, *133*, 4312–4320.

(5) Maeda, S.; Morokuma, K. Finding Reaction Pathways of Type A + B $\rightarrow$ X: Toward Systematic Prediction of Reaction Mechanisms. *J. Chem. Theory Comput.* **2011**, *7*, 2335–2345.

(6) `https://github.com/jensengroup/xtb_gaussian/tree/main`.

(7) Pracht, P.; Bohle, F.; Grimme, S. Automated exploration of the low-energy chemical space with fast quantum chemical methods. *Phys. Chem. Chem. Phys.* **2020**, *22*, 7169–7192.

(8) Zimmerman, P. M. Automated discovery of chemically reasonable elementary reaction steps. *J. Comp. Chem.* **2013**, *34*, 1385–1392.

(9) Zimmerman, P. Reliable Transition State Searches Integrated with the Growing String Method. *J. Chem. Theory Comput.* **2013**, *9*, 3043–3050.

(10) Rasmussen, M.; Jensen, J. Fast and automatic estimation of transition state structures using tight binding quantum chemical calculations. *PeerJ Physical Chemistry* **2020**, *2*, e15.

Table S7: Results for the 85 reactions curated as part of our study. A value of YES indicates that the corresponding product is successfully enumerated, whereas NO indicates that the product is not enumerated.

Reaction	Succesful	Reaction	Succesful	Reaction	Succesful
R1	NO	R4	YES	R7	NO
R2	NO	R5	YES	R8	YES
R3	NO	R12	YES	R13	NO
R14	YES	R17	NO	R18	YES
R19	YES	R21	YES	R23	YES
R24	YES	R25	YES	R26	NO
R27	YES	R28	NO	R29	YES
R30	YES	R31	YES	R32	YES
R33	YES	R34	YES	R36	YES
R37	YES	R38	YES	R39	NO
R40	YES	R41	YES	R42	YES
R43	YES	R44	YES	R45	YES
R46	YES	R47	YES	R48	YES
R49	YES	R50	YES	R51	YES
R52	NO	R53	YES	R55	NO
R56	YES	R57	YES	R58	YES
R59	YES	R60	YES	R61	YES
R62	YES	R63	YES	R64	YES
R65	YES	R66	NO	R67	YES
R69	NO	R70	NO	R71	YES
R72	NO	R73	YES	R74	YES
R75	YES	R76	NO	R77	YES
R78	YES	R79	YES	R80	NO
R81	NO	R82	NO	R83	NO
R84	YES	R85	NO	R86	YES
R88	YES	R89	NO	R91	YES
R92	YES	R97	YES	R98	YES
R99	YES	R100	YES	R101	YES
R102	NO				
